# The Influence of Thermal Curing Cycles on the Color Stability of Unfilled Polymethyl Methacrylate Teeth

**DOI:** 10.7759/cureus.23265

**Published:** 2022-03-17

**Authors:** Ram Basany, Vinod Bandela, Kulashekar Reddy Nandalur, Dileep Nag Vinnekota, Kiran Kumar Metta, Hari Kumar Mempally, Saraswathi Kanaparthi

**Affiliations:** 1 Prosthodontics, Sri Venkata Sai (SVS) Institute of Dental Sciences, Mahbubnagar, IND; 2 Prosthetic Dental Sciences, College of Dentistry, Jouf University, Sakaka, SAU; 3 Prosthetic Dental Sciences, College of Dentistry, Jazan University, Jazan, SAU; 4 Prosthodontics, Narayana Dental College and Hospital, Nellore, IND; 5 Conservative Dental Sciences, Ibn Sina National College for Medical Studies, Jeddah, SAU; 6 Prosthodontics, Aditya Dental College, Beed, IND; 7 Pedodontics and Preventive Dentistry, St. Joseph Dental College and Hospital, Eluru, IND

**Keywords:** polymethyl methacrylate (pmma), curing cycles, color stability, color difference, acrylic resin teeth

## Abstract

Purpose: The purpose of this study was to evaluate the influence of water bath polymerization cycles on the color stability of unfilled polymethyl methacrylate (PMMA) teeth.

Materials and methods: A total of 72 samples of unfilled PMMA were divided into two groups. Group 1 was subjected to a short curing cycle and group 2 to a long thermal curing cycle. Color measurements were done using a spectrophotometer and evaluated by the Commission Internationale de l’Eclairage (CIE) L*a*b* color system. Color difference (∆E) was calculated before and after thermal curing cycles and the data were statistically analyzed.

Results: Group 2 teeth exhibited the highest ΔE values and color change to long curing cycle with significant difference between them.

Conclusion: All the ΔE values were below 3.3, indicating that the color changes are not clinically perceptible.

## Introduction

Since time immemorial, the replacement of missing teeth has been a medical and cosmetic necessity for humankind. The reproducing ability of the shades and color blending in natural teeth to reproduce natural characteristics is easily achievable by the use of acrylics than with porcelains. The acrylic teeth have widespread use, making them an important adjunct to removable prostheses [1]. Despite having advantages like the ease of adjustment and chemical bonding to the resin denture base, they have disadvantages too, such as wearing and discoloration [2,3].

Color stability is the ability of a material to maintain its color in a given environment over a period of time. Various international standards have specified color stability as one of the important physical properties of acrylic resin teeth [4]. The technique for color determination can be manual/visual and instrumental [5]. There is the elimination of subjective errors with instrumental colorimeter in evaluating and assessing the color and color difference [6,7].

Acrylic resins do change in color as a result of intrinsic and extrinsic factors [8]. Exposure to physical and chemical conditions involving changes in temperature and humidity can result in intrinsic discoloration, whilst absorption and adsorption of fluids can promote extrinsic discoloration [9]. Leakage, aging degradation, and dehydration by stains are the other factors causing color changes in the acrylic denture teeth [10].

Austin and Basker found that polymerization in boiling water for a shorter period of time (30 minutes or less) encourages color instability to a greater extent, and yields seven times more residual monomer to short processing method than conventional curing cycle [11]. However, De Clerck suggested that a decrease in residual monomer and preserving the physical properties of resins can be achieved when microwave polymerization is followed. Other factors that cause discoloration of resins are the characterization of dentures and porosity as a result of overheating or lack of packing pressure during polymerization [12].

The color stability of polymers depends not only on the method of polymerization but also on the chemical characteristics of the resin material used, as suggested by Polyzois et al. [13]. Two suggested methods of polymerization of denture base resins are the long curing process at a uniform medium temperature and slow heating including a terminal boiling phase [14]. Assunção et al. studied the effect of polymerization methods and thermal cycling on the color change in 10 different acrylic resin denture teeth and concluded that the color difference (ΔE) values are within acceptable clinical limits for all the brands tested [9].

In the current study, we evaluated the color stability of unfilled polymethyl methacrylate (PMMA) teeth with two different thermal curing cycles.

## Materials and methods

Study design

**Figure 1:**
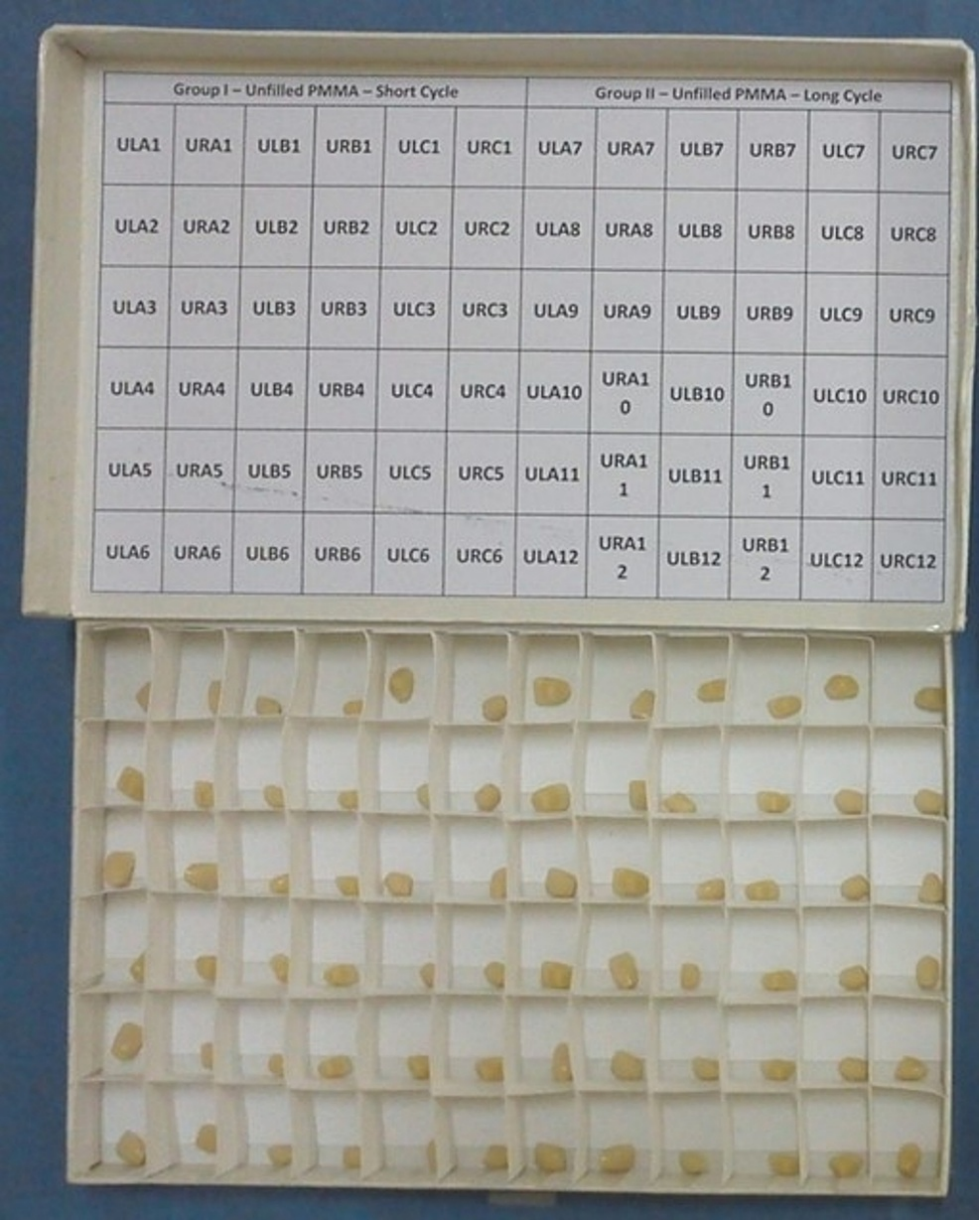
Grouping of unfilled polymethyl methacrylate teeth.

A total of 72 specimens of unfilled PMMA (Ivostar, Ivoclar Vivadent, Schaan, Liechtenstein) acrylic resin maxillary central incisor teeth were chosen for the study. The samples were grouped as group 1 (Gr-1) and group 2 (Gr-2), with 36 specimens in each group. They were sub-grouped into A and B groups. The teeth were labeled as upper right and upper left teeth for ease accordingly, i.e., ULA1 as upper left A1, URA1 as upper right A1, and so on (Figure 1).

Statistical analysis

The sample size was estimated by t-test, i.e., the difference between two independent means was chosen, using G*Power software (version 3.0). A minimum sample size of 72 (36 in each group) was found to be sufficient for an alpha of 0.05, power of 80%, and 0.67 as effect size.

Methodology

In each curing flask, six acrylic resin denture teeth of right and left sides were embedded separately with type III dental stone (Orthokal, LOT 00601, Kalabhai Karson Pvt. Ltd., Mumbai, India) and lab silicone (SilTech, LOT ML4139, Ivoclar Vivadent) in a metal flask (Varsity Flask #7, SS Products, Punjab, India) for polymerization in the water bath. The polymerization cycle, which was specific to that group, was carried out. Gr-1 samples were subjected to a short curing cycle (C-1, 74 oC for two hours with terminal boiling for one hour) and Gr-2 to a long curing cycle (C-2, 74 oC for eight hours with terminal boiling for one hour). After bench cooling, i.e., cooling the flasks for 24 hours, they were deflasked and specimens were retrieved, washed with distilled water, and dried with tissue paper. The specimens were then ready for evaluating the color.

Assessment

The measurement of color was done before and after thermal curing cycles with a spectrophotometer (ColorFlex® EZ, Hunter Associates Laboratory, Inc., Reston, VA), and color measurements were calculated by the Commission Internationale de l’Eclairage (CIE) L*a*b* system with standard illumination of D65. The color differences between the two measurements were calculated using the following formula [15]: ΔE = [(ΔL*)2 + (Δa*)2 + (Δb*)2]1/2.

A limit of ≤3.3 ΔE was considered clinically acceptable in the present study. The effect on the type of denture teeth and polymerization method on color stability (interactions among these factors) were analyzed by two-way repeated-measures analysis of variance (ANOVA).

## Results

**Figure 2:**
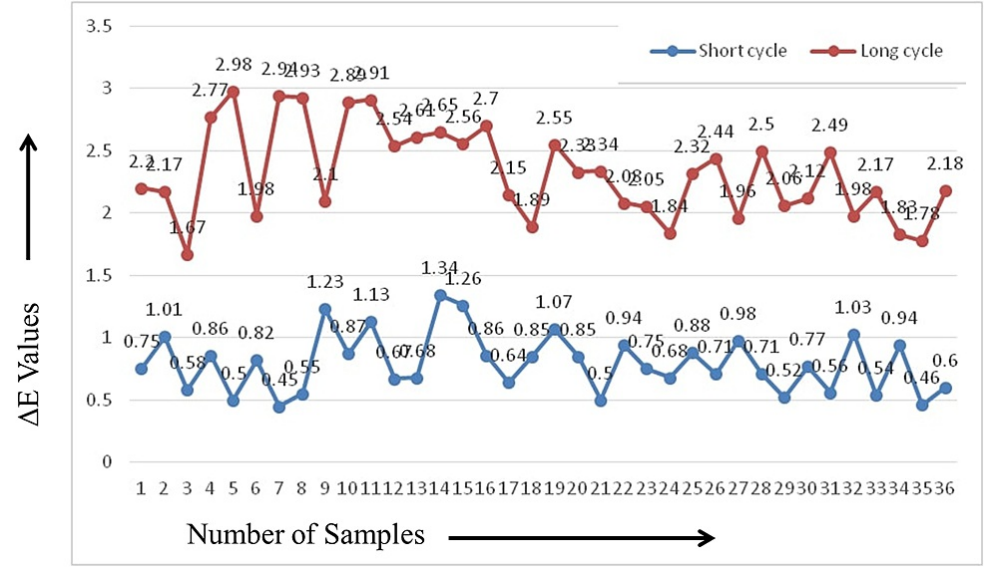
Line graph showing the ∆E of all samples in short and long curing cycle subgroups.

**Figure 3:**
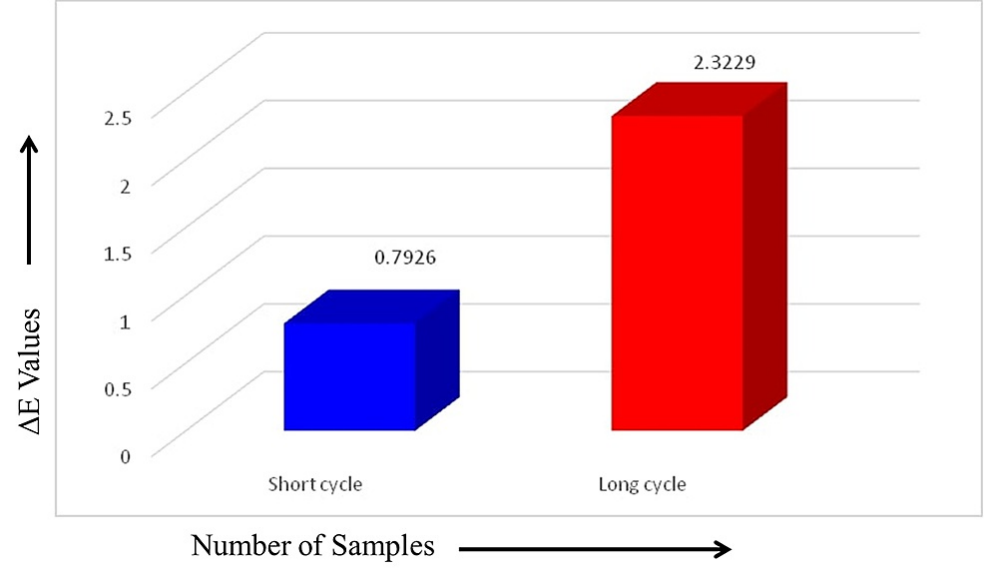
Mean ∆E of unfilled polymethyl methacrylate teeth among short and long curing cycles.

The mean and standard deviation of ΔE for the unfilled PMMA resin teeth at baseline measurements and after short and long curing cycles are as shown in Table 1. All the values obtained for ΔE in both curing cycles are less than 3.3. The samples in Gr-1 showed ∆E between 0.449 and 1.335, with a mean and standard deviation of 0.7926 ± 0.23501, while the samples in Gr-2 showed ∆E between 1.673 and 2.975, with a mean and standard deviation of 2.3229 ± 0.36839 (Figures 2, 3).

**Table 1:** Intergroup comparison of ∆E for unfilled polymethyl methacrylate teeth between short and long curing cycles.

	Cycle	N	Mean	Std. deviation	t-value	P-value
∆E	Short	36	0.7926	0.23501	−21.012	0.0014
	Long	36	2.3229	0.36839		

Comparison between the long and short curing cycles showed that the long curing cycle caused the greatest color change (2.3229 ± 0.36839). The difference between the two types of cycles is statistically significant (p = 0.0014), as shown in Table 1.

## Discussion

The classic material of choice for the fabrication of denture teeth is PMMA. Conventional unfilled PMMA, a material used by the majority of manufacturers to produce artificial teeth, has more than 50 years of proven clinical track record. During the manufacturing process, the molds were obtained by polymerization of a non-cross-linked linear polymer with a cross-linking agent monomer. The mixture typically consists of methyl methacrylate and dimethacrylate, in most cases, ethylene glycol dimethacrylate (EGDMA) (e.g., Ivostar, Ivoclar Vivadent) [6,8,9].

In the present study, the resin teeth were invested separately for long and short curing cycles in brass denture flasks using type-III dental stone and silicone lab putty. This was in accordance with the study by Shibayama et al., which emphasized the advantages of using silicone putty layer, i.e., the ease of application, improved cleanliness, creation of smoother denture surfaces, and greater ease in deﬂasking and polishing of the denture. They found the least positional changes regardless of the technique followed [16].

In this study, the specimens were subjected to two types of frequently adopted curing cycles: a short cycle and a long cycle. Whereas, a study by Honorez et al. adapted three different processing cycles [14]. Color evaluation can be performed visually or by using a spectrophotometer. The instrumental devices minimize subjective errors and are more accurate than visual measurement in color determination [3,5,7].

In the present study, the color of the samples was measured before and after thermal curing cycles with a spectrophotometer, and color changes were analyzed. In an in vitro study by Koksal and Dikbas, the color measurements were performed using a spectrophotometer and they concluded that the acrylic teeth showed a greater degree of color change than the porcelain teeth [3]. In another study by Ertaş et al., a colorimeter was used to measure the color of resin composite specimens [5].

In the current study, a spectrophotometer was used to measure the quantity of reflected light; color was then evaluated by the CIE L*a*b* color system, which was in agreement with the study of Rao et al. on tooth-colored restorative materials after bleaching, which used a similar technique for evaluating the color stability [17]. In the present study, color changes observed were below 2.97, expressive of lack of visible color change, which was similar to the study done by Assunção et al. [9]. The long curing cycle showed more color change. This may be due to the alteration of the organic matrix, inhibition, hydrolysis, breakdown of polymeric chains, and separation of cross-linking.

The limitations of the present study are as follows: a complete simulation of laboratory procedures for denture fabrication procedure was not carried out and the influence of different layers on the color stability was not studied, while the PMMA teeth of only one commercial brand were studied. More comparative studies need to be done on the effect of other polymerization methods such as microwave processing on different types of resin teeth.

## Conclusions

Within the limitations of the study, it was concluded that the color of denture teeth was affected by the polymerization methods, as there was a significant difference between short and long-cycle polymerization. Unfilled PMMA teeth showed the greatest color change to the long curing cycle than the short curing cycle. All ΔE values were below 3.3, indicative of the color change that is not clinically perceptible. As a long curing cycle gives optimal physical properties for the denture base, this method should be chosen for denture fabrication.
